# Optimization of Fast Dissolving Etoricoxib Tablets Prepared by Sublimation Technique

**DOI:** 10.4103/0250-474X.40335

**Published:** 2008

**Authors:** D. M. Patel, M. M. Patel

**Affiliations:** Department of Pharmaceutics and Pharmaceutical Technology, Shri Sarvajanik Pharmacy College, Mehsana - 384 001, India; 1 Shree S. K. Patel College of pharmaceutical Education & Research, Kherva - 382 711, India

**Keywords:** Fast dissolving tablets, etoricoxib, sublimation, menthol, factorial design, response surface plot

## Abstract

The purpose of this investigation was to develop fast dissolving tablets of etoricoxib. Granules containing etoricoxib, menthol, crospovidone, aspartame and mannitol were prepared by wet granulation technique. Menthol was sublimed from the granules by exposing the granules to vacuum. The porous granules were then compressed in to tablets. Alternatively, tablets were first prepared and later exposed to vacuum. The tablets were evaluated for percentage friability and disintegration time. A 3^2^ full factorial design was applied to investigate the combined effect of 2 formulation variables: amount of menthol and crospovidone. The results of multiple regression analysis indicated that for obtaining fast dissolving tablets; optimum amount of menthol and higher percentage of crospovidone should be used. A surface response plots are also presented to graphically represent the effect of the independent variables on the percentage friability and disintegration time. The validity of a generated mathematical model was tested by preparing a checkpoint batch. Sublimation of menthol from tablets resulted in rapid disintegration as compared with the tablets prepared from granules that were exposed to vacuum. The optimized tablet formulation was compared with conventional marketed tablets for percentage drug dissolved in 30 min (Q_30_) and dissolution efficiency after 30 min (DE_30_). From the results, it was concluded that fast dissolving tablets with improved etoricoxib dissolution could be prepared by sublimation of tablets containing suitable subliming agent.

The tablet is the most widely used dosage form because of its convenience in terms of self-administration, compactness, and ease in manufacturing. However, geriatric and pediatric patients experience difficulty in swallowing conventional tablets, which leads to poor patient compliance. To overcome this weakness, scientists have developed innovative drug delivery systems known as fast dissolving tablets. Their characteristic advantages such as administration without water, anywhere, anytime lead to their suitability to geriatric and pediatric patients. They are also suitable for the mentally ill, the bedridden and patients who do not have easy access to water. The benefits, in terms of patient compliance, rapid onset of action, increased bioavailability, and good stability make these tablets popular as a dosage form of choice in the current market^1^,^2^.

The fundamental principle used in the development of the fast-dissolving tablet is to maximize its pore structure. Researchers have evaluated spray dried materials^3^ and plastic materials^4^ for development of such tablets. Vacuum-drying^5^–^10^ and freeze-drying^11^–^14^ techniques have been tried by researchers to maximize the pore structure of tablet matrix. Freeze drying is cumbersome and yields a fragile and hygroscopic product. Therefore, a vacuum-drying technique was adopted in the present investigation after addition of a subliming agent to increase porosity of the tablets. It is likely that a porous hydrophilic matrix will easily pick up the disintegrating medium and break quickly.

Clinically, nonsteroidal antiinflammatory drugs (NSAIDs) are the most frequently prescribed by physicians for inflammatory disorders. NSAIDs exert their effect through inhibition of cyclooxygenase-II, the main form of isozyme associated with inflammation. But the simultaneous inhibition of cyclooxygenase-I and the resulting gastric and renal dysfunction limit their frequent use^15^. Etoricoxib is a cyclooxyginase-II (COX-II) selective NSAID used in the treatment of rheumatoid arthritis, osteoarthritis, postoperative dental pain, chronic low back pain, acute gout and primary dismenorrhoea^16^. The COX-I to COX-II selectivity ratio is higher than other COX-II inhibitors such as rofecoxib, valdecoxib and celecoxib^17^. Etoricoxib is practically insoluble in water and peak blood level reaches after 1 h of oral administration. The rate and extent of dissolution of the drug from any solid dosage form determines rate and extent of absorption of the drug. In the case of poorly water-soluble drugs, dissolution is the rate-limiting step in the process of drug absorption that in turns dependent on disintegration. The dissolution rate and bioavailability of poorly soluble drug from solid dosage form depend much on formulation additives and formulation characteristics. Thus, an attempt was made in the present investigation to prepare optimized fast dissolving tablets of etoricoxib using various sublimating materials.

## MATERIALS AND METHODS

Etoricoxib (EXB) was received as a gift sample from Sun Pharmaceuticals Ltd., Vadodara, India. Croscarmellose sodium (CCS), crospovidone (CRP) and sodium starch glycolate (SSG) were received as gift samples from Zydus Cadila Healthcare Ltd., Ahmedabad, India. Polyvinyl pyrrolidone K25 (PVP) was purchased from Loba Chemicals, Mumbai, India. Mannitol, camphor, menthol and ammonium bicarbonate were purchased from Laser Chemicals, Ahmedabad, India. All other ingredients used were of pharmaceutical grade.

### Preparation of etoricoxib tablets:

The raw materials were passed through a 100-mesh screen prior to mixing. EXB, aspartame, subliming material, intragranular fraction of disintegrant and mannitol were mixed using a glass mortar and pestle. All the ingredients were dry blended for 10 min and alcoholic solution of PVP was added to the mixture in a quantity just enough to bind the mass. The wet mass was passed through 30 mesh and the resulting granules (30 g) of 30/100-mesh screen were collected and vacuum dried at 65° for 24 h to facilitate sublimation of subliming materials. The granules were mixed with the extragranular fraction of crospovidone and the required proportion of fines (10%). The granules were lubricated with a blend containing talc (2%), magnesium stearate (1%) and sodium lauryl sulphate (0.5%). This uniformly mixed blend was compressed into tablets using flat face round tooling on a Rimek-I rotary tablet machine (Karnavati Eng. Pvt. Ltd, Ahmedabad). Sublimation was performed from tablets instead of granules at 65° in selected batches (A7 and F1 to F9 and check point). The composition of the preliminary and factorial batches is shown in Table 1 and 2, respectively.

**TABLE 1:** TABLET FORMULATION AND EVALUATION RESULTS OF PRELIMINARY TRIALS

Formulation Ingredients	A1	A2	A3	A4	A5	A6	A7
Etoricoxib (mg)	60	60	60	60	60	60	60
Ammonium bicarbonate (mg)	10	-	-	-	-	-	-
Camphor (mg)	-	10	-	-	-	-	-
Menthol (mg)	-	-	10	0	20	20	20
Aspartame (mg)	5	5	5	5	5	5	5
Crospovidone (mg)†	8	8	8	8	8	8	8
Colloidal silicon dioxide (mg)	-	-	-	-	-	2	2
Mannitol q. s. to--- (mg)	200	200	200	200	200	200	200
Disintegration time (s)	174	210	152	225	110	103	34
Friability (%)	0.36	0.21	0.47	0.15	0.52	0.33	0.35

All batches contained 10% polyvinylpyrrolidone in ethanol as a binder and 2% talc, 1% magnesium stearate and 0.5% sodium lauryl sulphate.

† Intragranular 50%; extragranular 50%. Subliming material was sublimed from granules in batches A1 to A6 and from tablets in batch A7

**TABLE 2:** FORMULATION AND EVALUATION OF BATCHES IN FULL FACTORIAL DESIGN

Batch Code	Variable levels in coded form†		DT ± SD (s)	F ± SD (%)
			
	X_1_	X_2_		
F1	−1	−1	232 ± 2.25	0.177 ± 0.014
F2	0	−1	55 ± 1.30	0.297 ± 0.013
F3	1	−1	47 ± 1.47	0.414 ± 0.011
F4	−1	0	220 ± 3.52	0.131 ± 0.016
F5	0	0	38 ± 2.03	0.263 ± 0.010
F6	1	0	32 ± 1.94	0.352 ± 0.017
F7	−1	1	196 ± 3.71	0.113 ± 0.013
F8	0	1	35 ± 2.37	0.254 ± 0.018
F9	1	1	22 ± 1.76	0.275 ± 0.012
Check point	−0.3	0.7	67 ± 2.13	0.197 ± 0.016

All batches contained 60 mg etoricoxib, 5 mg aspartame, 2 mg colloidal silicon dioxide, 10% polyvinylpyrrolidone in ethanol as a binder, 2% talc, 1% magnesium stearate, 0.5% sodium lauryl sulphate and mannitol q.s to 200 mg.

† X_1_ is amount of menthol, where, −1 = 0, 0 = 10 and 1 = 20 mg; X_2_ is amount of crospovidone, where, −1 = 6, 0 = 8 and 1 = 10 mg. DT indicates disintegration time; F, friability; and SD, standard deviation

### Evaluation of formulated tablets:

The crushing strength of the tablets was measured using a Monsanto hardness tester (Sheetal Scientific Industries, Mumbai, India). The friability of a sample of 20 tablets was measured using a Roche Friabilator (Electrolab, India). Twenty preweighed tablets were rotated at 25 rpm for 4 min. The tablets were then reweighed after removal of fines and the percentage of weight loss was calculated. For determination of disintegration time, one tablet was placed in each tube of disintegration apparatus (model ED2, Electrolab, India). Disintegration test was carried out using distilled water as a disintegrating media at 24±2°. The evaluation results of the preliminary and factorial batches are shown in Tables 1 and 2, respectively.

### Full factorial design:

A 3^2^ randomized full factorial design was adopted to optimize the variables. In this design 2 factors were evaluated, each at 3 levels, and experimental trials were performed at all 9 possible combinations^18^. The amounts of subliming agent, menthol (X_1_), and the amount of crospovidone (X_2_), were selected as independent variables. The disintegration time (DT) and percentage friability (%F) were selected as dependent variables.

### *In vitro* dissolution study and comparison with marketed tablets:

*In vitro* dissolution study was conducted using USP dissolution apparatus II (model TDT-06T, Electrolab, India) at 100 rpm; using 0.1 N HCL as dissolution media maintained at 37±0.5°. Samples were withdrawn at various time intervals, filtered through 0.45 μ membrane filter, diluted and assayed at 234 nm using a UV/Vis double beam spectrophotometer. The optimized tablet formulation (F8) was compared with conventional marketed tablets for percentage drug dissolved in 30 min (Q_30_) and dissolution efficiency^19^ after 30 min (DE_30_).

## RESULTS AND DISCUSSION

Water insoluble diluents such as microcrystalline cellulose and dicalcium phosphate were omitted from the study as they are expected to cause an unacceptable feeling of grittiness in the mouth. Among the soluble diluents, mannitol was selected as a diluent considering its advantages in terms of easy availability and negative heat of dissolution. The preliminary trials were conducted using 2% superdisintegrant (CCS, CRP and SSG) intragranularly and 2% extragranularly. Three batches were prepared using a single superdisintegrant, while the other three batches were prepared using an equal proportion of two disintegrants. Granules of mannitol and superdisintegrants were prepared by wet granulation technique using alcoholic solution of PVP (10% w/v) as a binder. The granules were lubricated and compressed into tablets on a rotary tablet machine. On the basis of the results obtained in the preliminary screening studies, the batch containing CRP showed the fastest disintegration. Hence, it was selected for further studies. PVP was used as a binder, considering its widespread applicability in the industry. Aspartame was included in the formulation as a sweetener to mask the bitter taste of EXB. Subliming agents such as ammonium bicarbonate, menthol and camphor were used to increase porosity of the tablets in the preliminary tablet formulations (batches A1 to A3). Menthol-containing tablets exhibited faster disintegration as compared with tablets containing ammonium bicarbonate and camphor. Batches A4 and A5 were prepared using menthol at different concentrations to study its effect on disintegration time. The sublimation time (5-10 h) depended on the amount of menthol present initially (0%, 5%, or 10%). Batch A5, containing 10% menthol, showed the least disintegrating time. The results shown in Table 1 indicate that concentration-dependent disintegration was observed in batches prepared using menthol as a subliming agent. The porous structure is responsible for faster water uptake; hence it facilitates wicking action of crospovidone in bringing about faster disintegration. Tablets with lower friability (≤0.5%) may not break during handling on machines and/or shipping. The use of a sublimating agent resulted in increased friability probably due to increased porosity. It was decided to incorporate colloidal silicon dioxide, extragranularly, at a level of 1% to decrease the friability of the tablets (batches A6 and A7). Addition of colloidal silicon dioxide resulted in appreciable decrease in friability and marginal decrease in disintegration time. Colloidal silicon dioxide helps to restore the bonding properties of the excipients^20^. In the first few attempts (A1 to A6), sublimation of subliming material was performed from granules prior to compression into tablets. Batches A1 to A6 showed good mechanical integrity, but the disintegration time was a little longer than the arbitrarily chosen value of less than 40 s. In Batch A7, sublimation was performed after compression rather than directly from granules. The results shown in Table 1 reveal that sublimation of menthol from tablets resulted in faster disintegration. The compaction process might have caused breakage of porous granules and subsequent reduction in porosity. The low value of disintegration time indicates that the porosity of tablets of batch A7 would be greater than batches A1 to A6. The granules required 3 h of vacuum drying, whereas the tablets required 10 h of vacuum drying. The longer drying time was required in the case of tablets probably because of the decreased surface area and porosity. In order to investigate the factors systematically, a factorial design was employed in the present investigation.

The amount of subliming agent (menthol, X_1_) and the superdisintegrant (crospovidone, X_2_) were chosen as independent variables in a 3^2^ full factorial design. A statistical model, Y = b_0_ + b_1_X_1_ + b_2_X_2_ + b_12_X_1_X_2_ + b_11_X_1_X_1_ + b_22_X_2_X_2_, incorporating interactive and polynomial terms was used to evaluate the responses; where Y is the dependent variable, b_0_ is the arithmetic mean response of the nine runs and b_i_ is the estimated coefficient for the factor X_i_. The main effects (X_1_ and X_2_) represent the average result of changing one factor at a time from its low to high value. The interaction terms (X_1_X_2_) show how the response changes when two factors are simultaneously changed. The polynomial terms (X_1_X_1_ and X_2_X_2_) are included to investigate nonlinearity. The DT and %F for the nine batches (F1 to F9) showed a wide variation (i.e., 22 to 232 s and 0.113% to 0.414%, respectively). The data clearly indicate that the DT and %F values are strongly dependent on the selected independent variables. The fitted equations (full and reduced) relating the responses DT and %F to the transformed factors are shown in Table 3. The polynomial equations can be used to draw conclusions after considering the magnitude of coefficient and the mathematical sign it carries (i.e., positive or negative). Table 4 shows the results of the analysis of variance (ANOVA), which was performed to identify insignificant factors^21^. The high values of correlation coefficient for DT and %F indicate a good fit i.e., good agreement between the dependent and independent variables. The equations may be used to obtain estimates of the response as a small error of variance was noticed in the replicates. The significance test for regression coefficients was performed by applying the student F test. A coefficient is significant if the calculated F value is greater than the critical value of F.

**TABLE 3:** SUMMARY OF RESULTS OF REGRESSION ANALYSIS

	b_0_	b_1_	b_2_	b_12_	b_11_	b_22_
Response (disintegration time)/coefficients						
FM	41.89	−91.167	−13.5	2.75	82.167	1.167
RM	42.67	−91.167	−13.5	-	82.167	-
Response (percentage friability)/coefficients								
FM	0.267	0.103	−0.041	−0.0188	−0.277	0.0063
RM	0.253	0.103	−0.041	-	-	-

FM indicates full model; and RM, reduced model

**TABLE 4:** CALCULATIONS FOR TESTING THE MODEL IN PORTIONS

	DF	SS	MS	F	*R*^2^	Fcal = 0.521
For disintegration time regression						
FM	5	64497	12899	407.95	0.998	Ftable = 9.55
RM	3	64464	21488	840.47	0.998	DF = (2, 3)
Error						
FM	3	94.86	31.62	-	-	
RM	5	127.83	25.57	-	-	
For % Friability regression									
FM	5	0.0772	0.0154	34.74	0.983	Ftable = 9.28
RM	2	0.0742	0.0371	51.14	0.944	DF = (3, 3)
Error						
FM	3	0.0013	0.0004	-	-	
RM	6	0.0043	0.0007	-	-	

DF indicates degree of freedom; SS, sum of squares; MS, mean of squares; F, Fischer's ratio; R^2^, regression coefficient; FM, full model; and RM, reduced model

**Fig. 1:**
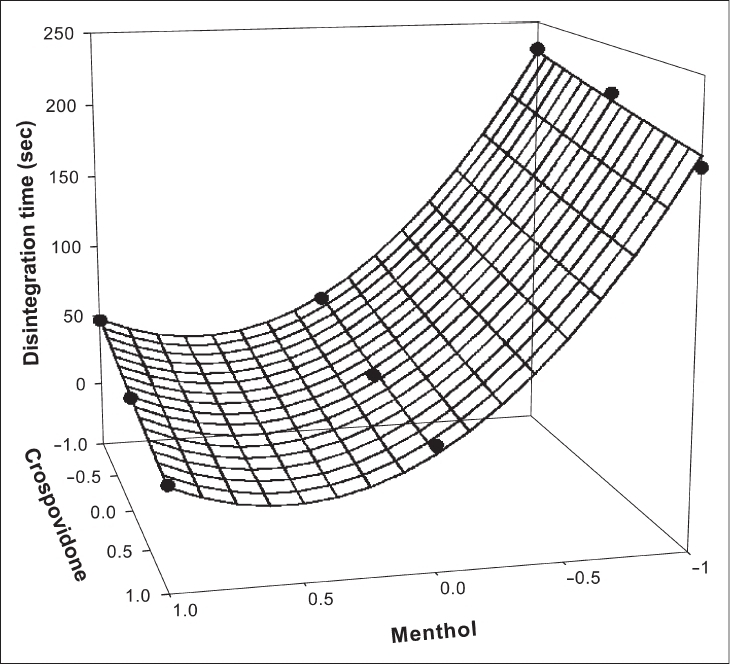
Response surface plot for disintegration time.

**Fig. 2:**
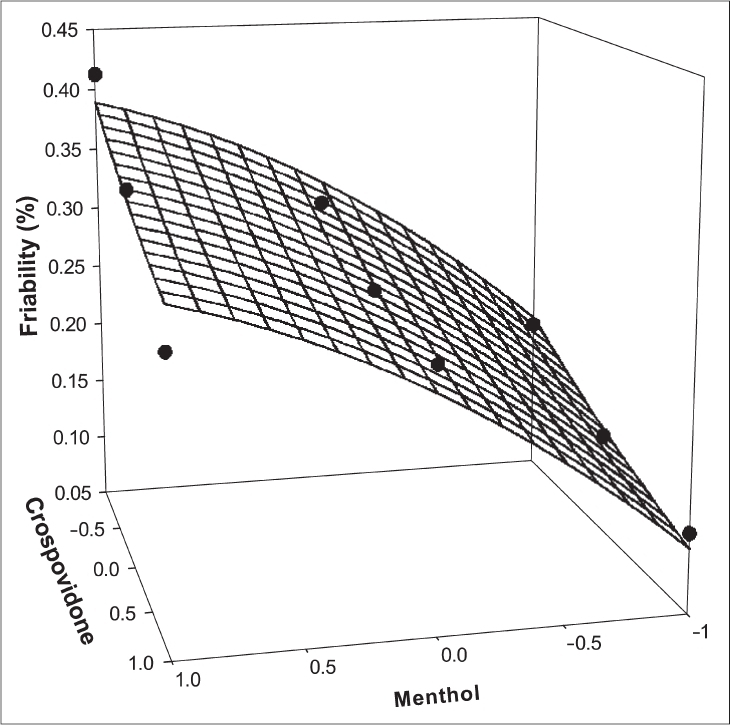
Response surface plot for percentage friability.

The significance level of coefficients b_12_ and b_22_ were found to be *p* = 0.4002 and 0.7883 respectively, hence they were omitted from the full model to generate the reduced model. The results of statistical analysis are shown in Table 3. The coefficients b_1_, b_2_, and b_11_ were found to be significant at *p* < 0.05; hence they were retained in the reduced model. The reduced model was tested in portions to determine whether the coefficients b_12_ and b_22_ contribute significant information for the prediction of DT or not. The results for testing the model in portions are shown in Table 4. The critical value of F for ∞ = 0.05 is equal to 9.55 (df = 2, 3). Since the calculated value (F = 0.521) is less than the critical value, it may be concluded that the interaction terms b_12_ and polynomial term b_22_ do not contribute significantly to the prediction of DT and therefore can be omitted from the full model. For drawing conclusions, response surface plot (figs. 1 and 2) should be used since one of the polynomial terms (b_11_) is also significant. The results of multiple linear regression analysis (reduced model) reveal that, on increasing the concentration of either menthol or crospovidone, a decrease in DT is observed; both the coefficients b_1_ and b_2_ bear a negative sign. When higher percentage of menthol is used, higher porosity is expected in the tablets. The water uptake and subsequent disintegration is thus facilitated. It is obvious that in the presence of higher percentage of crospovidone, wicking is facilitated. The fitted equation for full and reduced model relating the response was Y_DT_ = 41.89 − 91.167X_1_ − 13.5X_2_ + 82.167X_1_X_1_ + 1.167X_2_X_2_ + 2.75X_1_X_2_ and Y_DT_ = 42.67 − 91.167X_1_ − 13.5X_2_ + 82.167X_1_X_1_, respectively.

The significance level of coefficients b_11_, b_22_, and b_12_ were found to be *p* = 0.1604, 0.6995 and 0.1733 respectively, hence they were omitted from the full model to generate the reduced model. The results of statistical analysis are shown in Table 3. The coefficients b_1_ and b_2_ were found to be significant at *p* < 0.05; hence they were retained in the reduced model. The reduced model was tested in portions to determine whether the coefficients b_11_, b_22_, and b_12_ contribute significant information for the prediction of %F or not. The results for testing the model in portions are depicted in Table 4. The critical value of F for α = 0.05 is equal to 9.28 (df = 3, 3). Since the calculated value (F = 2.5) is less than the critical value, it may be concluded that the interaction term and polynomial terms do not contribute significantly to the prediction of %F. Hence, conclusions can be drawn considering the magnitude of the coefficient and the mathematical sign (positive or negative) it carries. An increase in the concentration of menthol leads to an increase in friability because the coefficient *b*_1_ bears a positive sign. When a higher percentage of menthol is used, more porous tablets are produced, which are mechanically weak. The increase in the concentration of crospovidone results in decreased friability values. Crospovidone is known to produce mechanically strong tablets. The fitted equation for full model and reduced model relating the response was Y_%F_ = 0.267 + 0.103X_1_ − 0.041X_2_ − 0.277X_1_X_1_ + 0.0063X_2_X_2_ − 0.019X_1_X_2_ and Y_%F_ = 0.267 + 0.103X_1_ − 0.041X_2_, respectively.

Figs. 1 and 2 show the plot of the amount of menthol (X_1_) and amount of crospovidone (X_2_) versus disintegration time and percentage friability, respectively. The plots were drawn using Sigma Plot Software (Jandel Scientific Software, San Rafael, CA). The plots demonstrate that both X_1_ and X_2_ affect the disintegration time and percentage friability. It was arbitrarily decided to select a batch of tablets that disintegrate in less than 40 s. Batches F5 (0,0), F6 (1,0), F8 (0,1) and F9 (1,1) fall in the acceptable criteria. The final selection is done after considering ease of manufacturing, cost, etc. In industry, the total time required for manufacturing a dosage form is of prime concern. When the variable X_1_ goes beyond 0 level (5%), vacuum drying time for complete sublimation increases hence batch F8 was selected as best batch. A checkpoint batch was prepared at X_1_ = −0.3 level and X_2_ = 0.7. From the reduced model, it is expected that the friability value of the checkpoint batch should be 0.207%, and the value of disintegration time should be 68 s. Table 2 indicates that the results are as expected. Thus, we can conclude that the statistical model is mathematically valid.

**Fig. 3:**
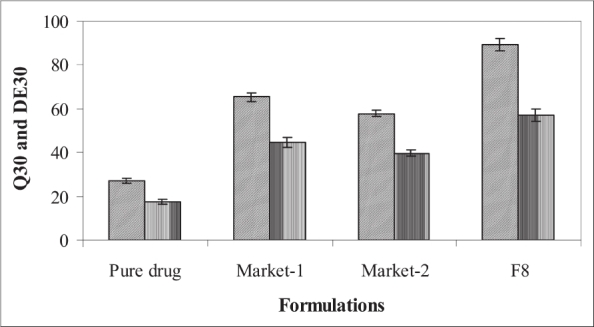
Comparative *in vitro* release and dissolution efficiency. DE_30_ (▒): Dissolution efficiency after 30 min; Q_30_ (▒): Cumulative % drug release in 30 min. Market 1: Torcoxia-60 from Torrent Pharmaceuticals Ltd., Indrad, Gujarat. Market 2: Etoxib-60 from Unichem Laboratories, Mumbai.

The batch F8 was compared with two marketed tablet formulations for *in vitro* drug release profile (Q_30_) and % dissolution efficiency (DE_30_). The values of Q_30_ and DE_30_ for F8 were higher than marketed tablets (Fig. 3) indicating superiority of the formulation F8. Therefore formulation F8 was considered better formulation with rapid and higher *in vitro* dissolution of etoricoxib.

The factorial batches were subjected to short-term stability studies at 40° and 75% RH for 3 mo. Samples withdrawn after 3 mo showed no significant change in appearance of the tablets, disintegration time and percentage friability.

The results of a 3^2^ full factorial design revealed that the amount of menthol and crospovidone significantly affect the dependent variables, disintegration time and percentage friability. It is thus concluded that by adopting a systematic formulation approach, an optimum point can be reached in the shortest time with minimum efforts. Sublimation technique would be an effective alternative approach compared with the use of more expensive adjuvants in the formulation of fast dissolving tablets with improved drug dissolution.
